# Association Between SGLT2 Inhibitor Use and Hepatocellular Carcinoma Risk in Type 2 Diabetes: A Systematic Review and Meta-Analysis †

**DOI:** 10.3390/biomedicines14051168

**Published:** 2026-05-21

**Authors:** Jing-Hong Hu, Ming-Ling Chang, Tung-Jung Huang, Nai-Jen Liu, Jui-Hsiang Tang

**Affiliations:** 1 Division of Gastroenterology and Hepatology, Yunlin Chang Gung Memorial Hospital, Yunlin County 638, Taiwan; donaldhuang@cgmh.org.tw; 2 College of Medicine, Chang Gung University, Taoyuan 333, Taiwan; minglingc@cgmh.org.tw; 3 Department of Gastroenterology and Hepatology, Linkou Chang Gung Memorial Hospital, Taoyuan 333, Taiwan; launaijn.tw@yahoo.com.tw; 4 Division of Gastroenterology and Hepatology, Department of Internal Medicine, Jen-Ai Hospital, Dali Branch, Taichung 412, Taiwan; ercp.tw@msa.hinet.net

**Keywords:** SGLT2 inhibitor, hepatocellular carcinoma, type 2 diabetes, meta-analysis, observational study, systematic review

## Abstract

**Background and Aims**: Type 2 diabetes mellitus is a recognized risk factor for hepatocellular carcinoma (HCC), particularly in the setting of metabolic dysfunction-associated steatotic liver disease (MASLD), chronic viral hepatitis, advanced fibrosis, and cirrhosis. Beyond hyperglycemia and insulin resistance, diabetic hepatocarcinogenesis is shaped by metabolic inflammation, lipotoxicity, oxidative stress, fibrogenic remodeling, and the cirrhosis-dysplasia-HCC continuum. Sodium-glucose cotransporter-2 inhibitors (SGLT2i) may influence several hepatometabolic pathways, but the epidemiologic evidence linking SGLT2i use to HCC risk remains heterogeneous. **Methods**: We conducted a systematic review and meta-analysis of observational studies evaluating SGLT2i exposure and incident HCC in adults with type 2 diabetes. PubMed, Embase, and the Cochrane Library were searched up to 15 March 2026. Adjusted time-to-event estimates were pooled using a restricted maximum likelihood (REML) random-effects model. The certainty of evidence was assessed using the GRADE framework and judged to be very low. **Results**: Six observational studies including 526,446 participants were included. SGLT2i exposure was associated with a lower observed risk of incident HCC (pooled HR 0.59, 95% CI 0.45–0.77), but between-study heterogeneity was substantial (I^2^ = 75.2%, τ^2^ = 0.074). The association remained directionally similar after exclusion of Huynh et al. (HR 0.61, 95% CI 0.45–0.81) and in a DPP-4 inhibitor-restricted active-comparator analysis (HR 0.60, 95% CI 0.39–0.92). However, the 95% prediction interval crossed the null (0.25–1.37), indicating that future comparable studies may plausibly show no protective association. **Conclusions**: SGLT2i exposure was associated with a lower observed risk of incident HCC across available observational studies. However, the certainty of evidence was judged to be very low, and substantial heterogeneity, comparator variation, mixed time-to-event estimands, residual confounding, and a prediction interval crossing the null preclude causal interpretation. These findings should be considered hypothesis-generating rather than practice-changing evidence and support further hepatology-oriented validation.

## 1. Introduction

Primary liver cancer remains a major global health burden. Recent GLOBOCAN-based estimates reported approximately 866,000 new liver cancer cases and 759,000 liver cancer deaths worldwide in 2022 [1]. HCC represents the dominant histologic subtype of primary liver cancer and most commonly arises in the setting of chronic liver injury, advanced fibrosis, and cirrhosis [2]. In parallel, diabetes has become an expanding global metabolic disorder; the International Diabetes Federation estimated that 589 million adults aged 20–79 years were living with diabetes worldwide in 2024 [3]. These converging epidemiologic trends create an increasingly large population at risk for MASLD, fibrosis progression, cirrhosis, and liver-related malignancy.

Type 2 diabetes mellitus is an established risk factor for HCC and may contribute to hepatocarcinogenesis through multiple intersecting pathways [4,5,6]. In diabetic liver disease, hepatocarcinogenesis rarely reflects a single mechanism. Chronic metabolic injury may promote insulin resistance, hyperinsulinemia, hepatic lipotoxicity, mitochondrial dysfunction, oxidative DNA damage, inflammatory cytokine activation, and hepatic stellate cell-mediated fibrogenesis. Over time, progressive fibrosis and cirrhotic nodular remodeling create a permissive hepatic microenvironment for dysplastic transformation and malignant progression. This fibrosis-cirrhosis-dysplasia-HCC continuum is particularly relevant in patients with type 2 diabetes, in whom MASLD, chronic viral hepatitis, alcohol-related liver disease, obesity, and other metabolic comorbidities may coexist and interact [2,6,7].

Sodium-glucose cotransporter-2 inhibitors are now widely used in type 2 diabetes because of their favorable cardiovascular and renal profiles [8,9,10]. Beyond glycemic control, these agents reduce body weight, improve insulin resistance, and may ameliorate hepatic steatosis, metabolic inflammation, oxidative stress, and fibrogenic signaling [11,12]. These hepatometabolic effects have prompted interest in whether SGLT2i exposure may be associated with lower HCC risk. However, biological plausibility alone is insufficient to infer chemopreventive efficacy, particularly when the available clinical evidence is derived from observational datasets.

Several real-world observational studies have examined the association between SGLT2i use and incident HCC, but their findings vary according to population selection, comparator therapy, baseline liver disease burden, follow-up duration, outcome ascertainment, and analytical approach [13,14,15,16,17,18]. Unlike broader reviews of major adverse liver outcomes or surrogate liver biomarkers, the present study focused specifically on incident HCC as a hard oncologic endpoint. We also explicitly evaluated comparator heterogeneity, time-to-event estimand differences, prediction intervals, sensitivity analyses, and certainty of evidence to provide a cautious hepatology-oriented synthesis of the available observational evidence.

## 2. Materials and Methods

### 2.1. Study Design and Registration

This study was designed as a systematic review and meta-analysis of observational studies evaluating the association between SGLT2 inhibitor exposure and incident hepatocellular carcinoma in adults with type 2 diabetes. The protocol was registered in PROSPERO (CRD420251090915), and the review was conducted in accordance with PRISMA 2020 principles [19].

### 2.2. Eligibility Criteria

Eligible studies enrolled adults with type 2 diabetes, evaluated exposure to any approved SGLT2 inhibitor, included a comparator group without SGLT2 inhibitor exposure or treated with alternative glucose-lowering therapies, and reported incident HCC with an adjusted effect estimate. Observational cohort studies, population-based database studies, and nested case–control studies were eligible. Reviews, editorials, case reports, conference abstracts without sufficient extractable data, animal studies, and studies without usable adjusted effect estimates were excluded.

### 2.3. Search Strategy and Study Selection

PubMed, Embase, and the Cochrane Library were searched from inception to 15 March 2026 using controlled vocabulary and free-text terms related to SGLT2 inhibitors, type 2 diabetes, hepatocellular carcinoma, and liver cancer. Two reviewers independently screened titles and abstracts, assessed full texts for eligibility, and resolved disagreements by discussion. Full database-specific search strings are provided in Supplementary Table S1. Records retrieved from the three databases were imported into a reference manager and de-duplicated before screening using automated matching followed by manual review of potential duplicate records. The relatively high proportion of duplicate records was attributable to the intentional use of highly sensitive, overlapping search strings across the three databases to ensure comprehensive capture of relevant literature. No additional records were identified through manual searching or other methods.

### 2.4. Data Extraction and Overlap Adjudication

Two reviewers independently extracted study characteristics, population features, exposure and comparator definitions, follow-up duration, HCC ascertainment methods, adjusted effect estimates, and covariates included in adjusted models. When multiple adjusted estimates were reported, the most fully adjusted overall estimate was preferred. When a study reported multiple eligible comparator arms, only one non-overlapping estimate was retained for the primary meta-analysis according to a prespecified comparator hierarchy. To evaluate potential population overlap, we compared each included study according to country or region, database source, enrollment period, population eligibility criteria, exposure definition, comparator framework, and outcome definition. When multiple potentially overlapping estimates were available, we retained a single estimate for the primary analysis according to the prespecified hierarchy favoring the most clinically relevant, most fully adjusted, and least overlapping comparison. Nevertheless, because patient-level data were unavailable, residual overlap among large database-driven studies, particularly those using federated electronic health record networks such as TriNetX, could not be definitively excluded.

### 2.5. Quality Assessment and Statistical Analysis

Methodological quality was assessed using the Newcastle–Ottawa Scale [20]. Studies with scores of 7–9 were considered high quality, 5–6 moderate quality, and 4 or lower low quality. Adjusted time-to-event estimates were pooled on the log scale using a restricted maximum likelihood (REML) random-effects model [21]. Because the number of eligible studies was small, conventional Cox HRs and competing-risk subdistribution HRs were pooled together on the log scale. This approach was statistically feasible but clinically imperfect. Cox HRs estimate cause-specific time-to-event associations, whereas subdistribution HRs incorporate the competing-risk structure and therefore answer a related but non-identical clinical question. This distinction is particularly important in older adults and patients with cirrhosis or advanced liver disease, in whom non-HCC mortality may represent a substantial competing event. Accordingly, the pooled estimate was interpreted as a pragmatic summary of adjusted time-to-event associations rather than as a single homogeneous causal estimand. One study [14] reported a confidence interval with pronounced log-scale asymmetry; the standard SE approximation [log(UCL) − log(LCL)]/(2 × 1.96) was applied for consistency with all other included studies.

Heterogeneity was assessed using Cochran’s Q, the I^2^ statistic [22], and τ^2^. A 95% prediction interval (PI) was calculated using the Student t distribution (df = k − 2) to appropriately reflect uncertainty in small-sample meta-analyses. Prespecified analyses included leave-one-out sensitivity analysis, a sensitivity analysis excluding Huynh et al., and an active-comparator analysis restricted to DPP-4 inhibitor comparator studies. Exploratory subgroup analyses were performed within prespecified strata. Formal between-subgroup statistical testing was not conducted because of the limited number of available studies. Formal meta-regression was not performed as a primary analysis because only six studies were available; with such a small number of studies, meta-regression is statistically underpowered and vulnerable to unstable estimates, ecological bias, and spurious associations. An exploratory bivariable random-effects meta-regression with comparator type (DPP-4 inhibitor vs. non-DPP-4 inhibitor) as the moderator is reported in Supplementary Table S5 with explicit instability warnings. Because fewer than 10 studies were available, formal assessment of small-study effects and funnel plot asymmetry was considered underpowered [23]. The certainty of evidence was evaluated using the GRADE framework. E-values were calculated as a sensitivity metric for unmeasured confounding [24] and were not used as a formal GRADE upgrading criterion. All statistical analyses were performed using R version 4.5.3 (R Foundation for Statistical Computing, Vienna, Austria) with the ‘metafor’ package version 4.6-0.

Generative AI language tools were used to assist with manuscript language editing and formatting; all AI-assisted outputs were critically reviewed, verified, and approved by all authors, who take full responsibility for the integrity of this work.

## 3. Results

### 3.1. Study Selection

**Figure 1:**
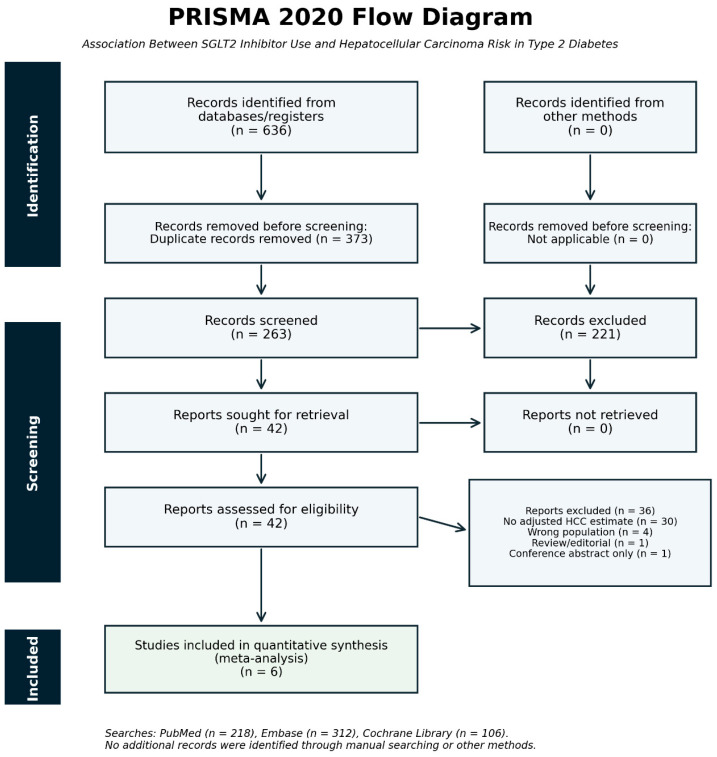
PRISMA 2020 flow diagram of study selection. Records were identified from PubMed, Embase, and the Cochrane Library (total *n* = 636). After removal of 373 duplicate records, 263 records underwent title/abstract screening; 42 reports were sought for retrieval, 0 were not retrieved, 42 full-text reports were assessed for eligibility, 36 were excluded, and 6 studies were included in the systematic review and quantitative synthesis.

A total of 636 records were identified through searches of PubMed, Embase, and the Cochrane Library. After duplicate removal, 263 records remained for title and abstract screening. Forty-two articles underwent full-text review, of which 36 were excluded and 6 met the eligibility criteria for inclusion in the primary quantitative synthesis. Among the 36 full-text articles excluded, the most common reason was absence of an adjusted HCC estimate (*n* = 30), followed by wrong population (*n* = 4), review/editorial article (*n* = 1), and conference abstract only (*n* = 1). The study selection process is summarized in Figure 1.

### 3.2. Study Characteristics

The six included studies were published between 2023 and 2026 and collectively enrolled 526,446 participants [13,14,15,16,17,18]. Key study characteristics are summarized in Table 1, and the study-level extraction sheet is provided in Supplementary Table S2. The evidence base comprised nationwide or territory-wide retrospective cohort studies, one US/Korea multi-institutional electronic health-record cohort, and one TriNetX-based retrospective propensity score-matched cohort. Comparator structures differed across studies and included DPP-4 inhibitor comparators, broader non-SGLT2i antidiabetic comparators, and, in one study, metformin monotherapy versus metformin plus SGLT2 inhibitor dual therapy. The Huynh study specifically enrolled patients with type 2 diabetes and cirrhosis and compared metformin monotherapy with dual metformin and SGLT2 inhibitor therapy over 5 years, with HCC occurrence as one of the predefined outcomes [18].

### 3.3. Primary Meta-Analysis

**Figure 2:**
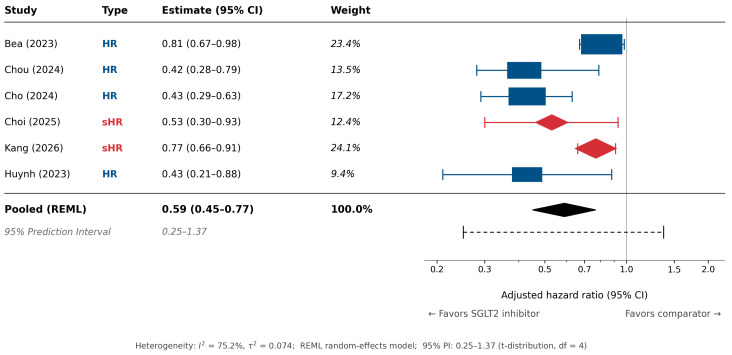
Forest plot of adjusted time-to-event estimates for the association between SGLT2 inhibitor use and incident hepatocellular carcinoma risk. Studies by Choi (2025) and Kang (2026) [16,17], shown as red diamonds, contributed subdistribution hazard ratios (sHR) from competing-risk Fine-Gray models; the remaining four studies Bea (2023) [13], Chou (2024) [14], Cho (2024) [15] and Huynh (2023) [18], shown as blue squares, contributed conventional Cox hazard ratios (HR). The pooled estimate was calculated using a restricted maximum likelihood (REML) random-effects model, and the dashed line denotes the 95% prediction interval (0.25–1.37).

Across six studies contributing adjusted time-to-event estimates, SGLT2 inhibitor exposure was associated with a lower observed risk of incident hepatocellular carcinoma, with a pooled estimate of 0.59 (95% CI 0.45–0.77) under a REML random-effects model. Between-study heterogeneity was substantial (I^2^ = 75.2%, τ^2^ = 0.074), indicating that the pooled estimate should be interpreted as a central summary across heterogeneous clinical and analytic contexts. The 95% prediction interval ranged from 0.25 to 1.37 (Student t distribution, df = 4), crossing the null and suggesting that a future study could plausibly show no protective effect. Therefore, although the pooled estimate suggested a lower observed HCC risk, the substantial between-study heterogeneity and prediction interval crossing the null indicate that this association may not be generalizable across all clinical settings, comparator frameworks, or liver disease populations. The forest plot is shown in Figure 2.

### 3.4. Sensitivity Analyses

The leave-one-out analysis showed that the pooled estimate remained below the null across all iterations, ranging from 0.53 to 0.64 depending on the excluded study. Exact leave-one-out outputs are provided in Supplementary Table S3 and Supplementary Figure S1.

Given the distinct exposure structure of Huynh et al., we performed a sensitivity analysis excluding that study. After exclusion of Huynh et al., the pooled estimate was 0.61 (95% CI 0.45–0.81; I^2^ = 78.7%), indicating that the inverse association remained directionally similar when restricted to studies more directly comparing SGLT2 inhibitor exposure against alternative antidiabetic strategies.

An active-comparator sensitivity analysis restricted to studies using DPP-4 inhibitor comparators [13,14,16] yielded a pooled estimate of 0.60 (95% CI 0.39–0.92; I^2^ = 67.4%). A consolidated summary of sensitivity and subgroup analyses is provided in Supplementary Table S4. An exploratory bivariable meta-regression with comparator type as the moderator (Supplementary Table S5) showed no statistically significant effect modification by comparator type (β = +0.070, 95% CI −0.538 to +0.677, *p* = 0.822; pseudo R^2^ = 0.0%); this exploratory analysis is reported with explicit small-sample instability warnings and should not be used to infer comparator-specific effect modification.

### 3.5. Exploratory Subgroup Analyses

In exploratory subgroup analyses, the pooled estimate in Asian cohorts was 0.61 (95% CI 0.44–0.86; I^2^ = 85.0%), whereas the pooled estimate in non-Asian or multi-institutional cohorts was 0.49 (95% CI 0.31–0.76; I^2^ = 0%). Similarly, chronic liver disease-enriched cohorts showed a pooled estimate of 0.56 (95% CI 0.36–0.86; I^2^ = 74.6%), whereas general type 2 diabetes cohorts showed a pooled estimate of 0.60 (95% CI 0.39–0.92; I^2^ = 67.4%). These subgroup findings, summarized in Table 2 and Table S4, should be interpreted descriptively given the limited number of studies.

All subgroup findings should be interpreted descriptively because of the limited number of included studies and the persistence of between-study heterogeneity. These subgroup findings should not be interpreted as evidence of definitive subgroup-specific effects. Because several strata included only two or three studies, the estimates are statistically unstable, and formal biological or clinical inferences from these subgroup comparisons should be avoided. In particular, the non-Asian or multi-institutional subgroup comprises only two studies (k = 2); the REML estimate of tau^2^ in k = 2 is inherently unstable and I^2^ = 0.0% likely reflects a boundary solution rather than true homogeneity. Formal between-subgroup inference was not emphasized because statistical power was limited. Estimates were pooled using REML random-effects models. Note that the “General type 2 diabetes cohorts” subgroup comprises the exact same three studies ([13,14,16]) as the DPP-4 inhibitor-restricted active-comparator analysis. This is an empirical coincidence in the current literature: all three studies that used a general T2DM population also happened to use a DPP-4 inhibitor comparator, hence the identical pooled estimate and I^2^ value across these two conceptually distinct analytical dimensions.

### 3.6. Risk of Bias and Certainty of Evidence

The methodological quality of the included studies was assessed using the Newcastle–Ottawa Scale (Table 3). Overall, the included studies were of high methodological quality (scores 7–9). Because fewer than 10 studies were available, formal assessment of publication bias and funnel plot asymmetry was considered underpowered [23], and publication bias could neither be confirmed nor excluded.

Using the GRADE framework, the overall certainty of evidence for the primary outcome was judged to be very low. Certainty started at low because all included studies were observational and was further downgraded for inconsistency and imprecision. E-values were reported separately as a sensitivity metric for unmeasured confounding and were not used as a formal GRADE upgrading criterion.

## 4. Discussion

In this systematic review and meta-analysis of observational studies, SGLT2i exposure was associated with a lower observed risk of incident HCC in adults with type 2 diabetes. However, this association should be interpreted as a hypothesis-generating observational signal rather than evidence of definitive chemopreventive efficacy. The main contribution of this study is not to establish causality, but to synthesize the emerging HCC-specific pharmacoepidemiologic evidence while explicitly highlighting heterogeneity, comparator variation, time-to-event estimand differences, and very low certainty of evidence.

The substantial heterogeneity deserves central emphasis. Although the pooled estimate was below the null, the 95% prediction interval crossed 1.0, indicating that future comparable studies may plausibly show no inverse association. This finding limits external generalizability and suggests that the pooled HR should not be interpreted as a universally transportable treatment effect. Rather, the summary estimate should be viewed as the central tendency of a heterogeneous observational evidence base spanning different regions, comparator drugs, liver disease backgrounds, outcome definitions, and analytic frameworks.

Comparator selection may represent one of the dominant sources of clinical heterogeneity across observational SGLT2i-HCC studies. Studies using DPP-4 inhibitor comparators approximate an active-comparator framework more closely than studies using broad non-SGLT2i comparators or metformin monotherapy. In particular, comparisons between metformin monotherapy and metformin plus SGLT2i dual therapy do not isolate an SGLT2i class effect in the same way as an incident new-user active-comparator design. For this reason, the overall pooled analysis should be interpreted as a broad synthesis of available observational associations, whereas the DPP-4 inhibitor-restricted analysis provides a more clinically coherent but still limited sensitivity estimate.

The mixture of conventional HRs and competing-risk subdistribution HRs also limits interpretability. These effect measures are mathematically poolable on the log scale, but they are not identical clinical estimands. Conventional Cox HRs focus on cause-specific time-to-event associations, whereas subdistribution HRs estimate effects on the cumulative incidence function in the presence of competing events. This distinction is particularly relevant in patients with cirrhosis, advanced liver disease, older age, or high non-HCC mortality risk. Therefore, the pooled estimate should be understood as a pragmatic summary of adjusted time-to-event associations rather than a single homogeneous causal parameter.

The observed inverse association is biologically plausible but should not be overinterpreted. Prior epidemiologic and mechanistic literature supports links among type 2 diabetes, MASLD, chronic inflammation, fibrosis progression, and HCC risk [2,4,5,6,7]. SGLT2 inhibitors may improve insulin resistance, reduce body weight and visceral adiposity, attenuate hepatic steatosis, and modulate inflammatory, fibrogenic, and oxidative pathways implicated in hepatocarcinogenesis [11,12]. Nevertheless, biological plausibility should be interpreted as supportive context rather than proof of chemoprevention. A recent meta-analysis by Mantovani et al. pooled active-comparator new-user observational cohorts and reported an association between SGLT2 inhibitor use and lower risk of broader major adverse liver-related outcomes, a composite that included hepatic decompensation, HCC, liver transplantation, and liver-related death [25]. Our study differs by focusing specifically on incident HCC as a hard oncologic endpoint and by explicitly adjudicating comparator structure, HR/sHR estimands, prediction intervals, and certainty of evidence.

Residual confounding remains a major limitation despite the use of propensity score matching, weighting, or multivariable adjustment in the included studies. SGLT2i users may differ from comparator patients in socioeconomic status, insurance coverage, healthcare access, liver imaging frequency, HCC surveillance intensity, alcohol consumption, medication adherence, health literacy, and health-seeking behavior. These unmeasured or incompletely measured factors could exaggerate the apparent inverse association if SGLT2i users were healthier, more adherent, or more consistently engaged with medical care than comparator patients. Accordingly, E-values should be interpreted only as a sensitivity metric for unmeasured confounding, not as proof that residual confounding has been eliminated.

Immortal time bias and prevalent user bias may also have influenced the pooled estimate. In studies without a clear incident new-user active-comparator design, patients classified as SGLT2i users may have had to remain alive and HCC-free long enough to initiate therapy, creating immortal person-time. Similarly, prevalent users may represent a selected group of patients who tolerated therapy and survived without early adverse outcomes. Both mechanisms could exaggerate an apparent protective association. Future studies should therefore prioritize incident new-user active-comparator designs with clear time-zero alignment and harmonized follow-up definitions.

From a hepatopathological perspective, the potential link between SGLT2i exposure and HCC risk must be interpreted within the broader hepatic microenvironment. In diabetic liver disease, chronic lipotoxic injury, oxidative stress, inflammatory cytokine activation, and hepatic stellate cell-driven fibrogenesis may promote progressive architectural remodeling. Once advanced fibrosis or cirrhosis develops, regenerative nodules, altered vascular architecture, immune dysregulation, and dysplastic transformation may contribute to the cirrhosis-dysplasia-HCC continuum. However, the included studies could not uniformly harmonize HBV, HCV, MASLD, alcohol-related liver disease, fibrosis stage, cirrhosis status, antiviral treatment exposure, or histopathologic confirmation of HCC. These limitations substantially constrain biological interpretation of the pooled association.

Geographic generalizability is also limited. Most available data were derived from East Asian populations or predominantly US-based multi-institutional electronic health record networks. The findings may therefore not be fully generalizable to Europe, Africa, Latin America, South Asia, or regions with different distributions of HBV, HCV, MASLD, alcohol-related liver disease, diabetes treatment access, and HCC surveillance practices.

Clinically, the current evidence should not alter routine hepatology or diabetes practice. SGLT2 inhibitors should continue to be prescribed according to established cardiometabolic, renal, and glycemic indications, rather than specifically for HCC prevention. The observed inverse association may support further hepatology-oriented research, particularly in patients with MASLD, advanced fibrosis, compensated cirrhosis, or chronic viral hepatitis, but it does not establish SGLT2i therapy as a chemopreventive intervention.

This study has several limitations. First, all included data were observational, and causal inference remains limited. Second, comparator heterogeneity complicates interpretation, because DPP-4 inhibitor comparators, broad non-SGLT2i comparators, and metformin monotherapy comparators represent clinically distinct reference frameworks. Third, baseline liver disease status was heterogeneous, with included populations spanning general type 2 diabetes, FLD/CVH-enriched cohorts, viral hepatitis cohorts, and cirrhosis cohorts. Fourth, mixed use of conventional HRs and competing-risk subdistribution HRs introduced estimand-level heterogeneity. Fifth, HCC ascertainment methods differed across studies and may have included registry-based diagnoses, ICD coding systems, imaging-based criteria, or histopathologic confirmation with varying degrees of validation. Sixth, potential population overlap across large database-driven studies, including TriNetX-based cohorts, could not be completely excluded. Seventh, residual confounding by socioeconomic status, surveillance intensity, alcohol exposure, medication adherence, healthcare access, and health-seeking behavior remains possible. Eighth, not all studies explicitly adopted incident new-user active-comparator designs, leaving potential immortal time bias and prevalent user bias. Ninth, individual SGLT2 inhibitors may differ in pharmacologic and hepatic effects, but drug-specific estimates were unavailable. Tenth, the evidence base was geographically concentrated in East Asia and predominantly US-based EHR networks. Finally, because fewer than 10 studies were available, publication bias could not be reliably assessed; the exploratory comparator-type meta-regression was necessarily underpowered and should not be interpreted as definitive source-of-heterogeneity testing.

Future research should prioritize hepatology-specific directions including incident new-user active-comparator designs; stratified analyses in patients with MASLD, advanced fibrosis, and compensated cirrhosis; etiology-specific analyses across HBV, HCV, MASLD, and alcohol-associated liver disease; harmonized HCC ascertainment; drug-specific analyses comparing individual SGLT2 inhibitors; and interaction analyses with antiviral therapy in chronic viral hepatitis.

## 5. Conclusions

SGLT2i exposure was associated with a lower observed risk of incident HCC in adults with type 2 diabetes across currently available observational studies. However, the certainty of evidence was very low, and the pooled estimate was limited by substantial heterogeneity, comparator variation, mixed time-to-event estimands, residual confounding, possible immortal time and prevalent user bias, and a prediction interval crossing the null. These findings should therefore be interpreted as hypothesis-generating rather than practice-changing evidence. SGLT2 inhibitors should not be prescribed specifically for HCC prevention on the basis of the current evidence alone. Further hepatology-oriented studies using incident new-user active-comparator designs, harmonized HCC ascertainment, etiology-specific liver disease stratification, and drug-specific analyses are warranted.

## Figures and Tables

**Table 1:** Characteristics of included observational studies.

Study	Region	Data Source	Design	Population	Comparator Retained	Effect Measure	Adjusted Estimate (95% CI)	Follow-Up	Outcome
Bea (2023) [13]	Korea	Korean national claims	Retrospective cohort	T2DM	DPP-4 inhibitor	HR	0.81 (0.67–0.98)	Median~3 y	Incident HCC
Chou (2024) [14]	Hong Kong	Population-based database	Retrospective cohort	T2DM	DPP-4 inhibitor	HR	0.42 (0.28–0.79)	Longitudinal	Incident HCC
Cho (2024) [15]	Korea	Korean HIRA	Retrospective cohort	FLD + T2DM; CVH subgroup	Non-SGLT2i comparator	HR	0.43 (0.29–0.63)	Longitudinal	Incident HCC
Choi (2025) [16]	US/Korea	MGB-Asan EHRs	Retrospective active-comparator cohort	T2DM	DPP-4 inhibitor retained	sHR	0.53 (0.30–0.93)	Median 3.9 y	Incident HCC
Kang (2026) [17]	Korea	Korean nationwide claims	Retrospective cohort	Viral hepatitis + T2DM	Non-SGLT2i comparator	sHR	0.77 (0.66–0.91)	Longitudinal	Incident HCC
Huynh (2023) [18]	TriNetX/ multi-institutional	TriNetX Research Network	PS-matched retrospective cohort	T2DM + cirrhosis	Metformin monotherapy	HR	0.43 (0.21–0.88)	5 y	HCC occurrence

Abbreviations: HR, hazard ratio; sHR, subdistribution hazard ratio (from competing-risk Fine-Gray models); T2DM, type 2 diabetes mellitus; HCC, hepatocellular carcinoma; FLD, fatty liver disease; CVH, chronic viral hepatitis; EHR, electronic health record; PS, propensity score. All HRs are adjusted estimates from the source studies. Note: Overlap was adjudicated by country, data source, health-system setting, and enrollment period; no confirmed duplicate patient population was identified across the retained primary contrasts.

**Table 2:** Exploratory subgroup analyses of adjusted time-to-event estimates.

Subgroup	Studies	Pooled Estimate (95% CI)	I^2^ (%)
Overall	6	0.59 (0.45–0.77)	75.2
Asian cohorts	4	0.61 (0.44–0.86)	85.0
Non-Asian or multi-institutional cohorts	2	0.49 (0.31–0.76)	0.0
Chronic liver disease-enriched cohorts	3	0.56 (0.36–0.86)	74.6
General type 2 diabetes cohorts	3	0.60 (0.39–0.92)	67.4

**Table 3:** Methodological quality assessment using the Newcastle–Ottawa Scale.

Study	Selection	Comparability	Outcome	NOS Total	Overall Quality
Kang (2026) [17]	4	2	3	9	High
Chou (2024) [14]	4	2	2	8	High
Cho (2024) [15]	4	2	2	8	High
Choi (2025) [16]	4	2	3	9	High
Bea (2023) [13]	3	2	2	7	High
Huynh (2023) [18]	3	2	2	7	High

NOS categories: 7–9, high quality; 5–6, moderate quality; 4 or lower, low quality.

## Data Availability

The data supporting the findings of this study were extracted from published primary studies. The analysis dataset and R code used for the present meta-analysis are available from the corresponding author upon reasonable request.

## Supplementary Materials

The following supporting information can be downloaded at: https://www.mdpi.com/article/10.3390/biomedicines14051168/s1, Figure S1: Leave-one-out sensitivity analysis of the pooled association between SGLT2 inhibitor use and incident hepatocellular carcinoma risk. Each row shows the pooled hazard ratio recalculated after sequential omission of one study from the six-study primary dataset; the overall pooled estimate from the full model is shown for reference. All analyses used a restricted maximum likelihood (REML) random-effects model; Table S1: Full database-specific search strategies; Table S2: Study-level extraction sheet; Table S3: Leave-one-out sensitivity analysis results (REML random-effects model); Table S4: Sensitivity and subgroup analyses (REML random-effects model); Table S5: Exploratory bivariable random-effects meta-regression with comparator type as moderator (REML); Supplementary File S1: PRISMA 2020 Checklist Page references correspond to the revised clean manuscript included in this package.
