# Supplementary material for: Association Between SGLT2 Inhibitor Use and Hepatocellular Carcinoma Risk in Type 2 Diabetes: A Systematic Review and Meta-Analysis
Source: Biomedicines. 2026 May 21;14(5):1168. doi: 10.3390/biomedicines14051168 (PMC13204993; doi:10.3390/biomedicines14051168)
Supplement: Supplementary file 1 [file biomedicines-14-01168-s001.zip › Supplementary File S1_PRISMA_Checklist_v9_0_FINAL.pdf]

Supplementary File S1. PRISMA 2020 Checklist Page references correspond to the revised clean manuscript included in this package.

Section / Topic	Item	Checklist item (abbrev.)	Manuscript page(s)
Title	1	Identify report as a systematic review/meta-analysis	1
Abstract	2	Structured abstract	1
Introduction	3	Rationale	2
Introduction	4	Objectives	2
Methods	5	Eligibility criteria	3
Methods	6	Information sources	3
Methods	7	Search strategy	3; Supplementary Table S1
Methods	8	Selection process	3
Methods	9	Data collection process	3
Methods	10	Data items	3
Methods	11	Study risk of bias assessment	3
Methods	12	Effect measures	3
Methods	13	Synthesis methods	3-4
Methods	14	Reporting bias assessment	4
Methods	15	Certainty assessment	4
Results	16	Study selection	4
Results	17	Study characteristics	4
Results	18	Risk of bias in studies	5
Results	19	Results of individual studies	5; Figure 2; Table 1
Results	20	Results of syntheses	4-5; Table 3; Supplementary Figure S1
Results	21	Reporting biases	5
Results	22	Certainty of evidence	5
Discussion	23	Discussion and limitations	5-6

Other	24	Registration and protocol	3; Title Page
Other	25	Support	Title Page
Other	26	Competing interests	Title Page
Other	27	Availability of data/materials	Title Page; Cover Letter
