# Supplementary material for: Association Between SGLT2 Inhibitor Use and Hepatocellular Carcinoma Risk in Type 2 Diabetes: A Systematic Review and Meta-Analysis
Source: Biomedicines. 2026 May 21;14(5):1168. doi: 10.3390/biomedicines14051168 (PMC13204993; doi:10.3390/biomedicines14051168)
Supplement: Supplementary file 1 [file biomedicines-14-01168-s001.zip › Supplementary_Figure_S1_v9_0_FINAL.pdf]

Supplementary Figure S1. Leave-one-out sensitivity analysis of the pooled association between SGLT2 inhibitor use and incident hepatocellular carcinoma risk. Each row shows the pooled hazard ratio recalculated after sequential omission of one study from the six-study primary dataset; the overall pooled estimate from the full model is shown for reference. All analyses used a restricted maximum likelihood (REML) random-effects model.

Supplementary Figure S1. Leave-one-out sensitivity analysis

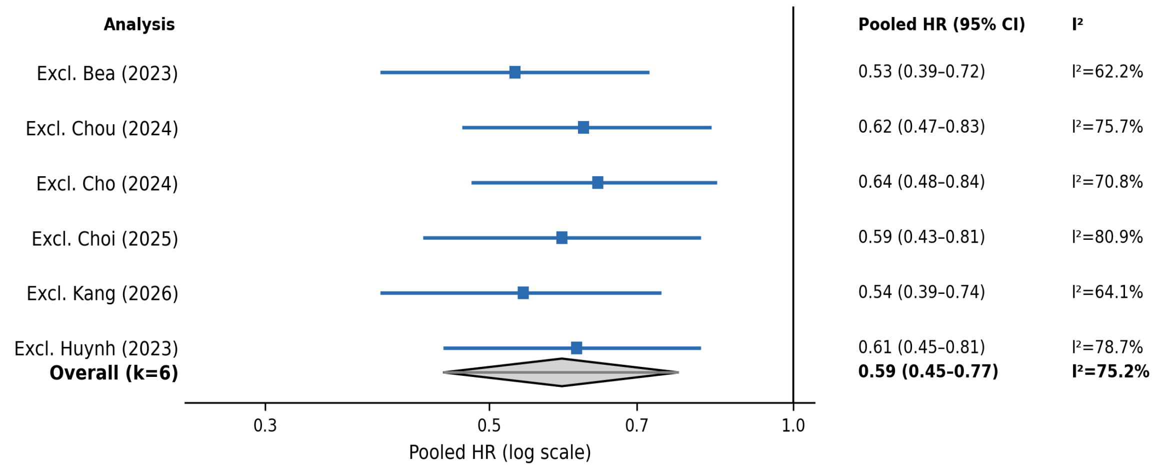

Squares represent pooled estimates after sequential omission of one study; the diamond represents the full six-study model.
