# Supplementary material for: Association Between SGLT2 Inhibitor Use and Hepatocellular Carcinoma Risk in Type 2 Diabetes: A Systematic Review and Meta-Analysis
Source: Biomedicines. 2026 May 21;14(5):1168. doi: 10.3390/biomedicines14051168 (PMC13204993; doi:10.3390/biomedicines14051168)
Supplement: Supplementary file 1 [file biomedicines-14-01168-s001.zip › Supplementary_Table_S1_Search_Strategies_v9_0_FINAL.pdf]

Supplementary Table S1. Full database-specific search strategies

PubMed

```
((SGLT2 inhibitor*[Title/Abstract]) OR sodium-glucose cotransporter-2
inhibitor*[Title/Abstract] OR dapagliflozin[Title/Abstract] OR
empagliflozin[Title/Abstract] OR canagliflozin[Title/Abstract] OR
ertugliflozin[Title/Abstract] OR ipragliflozin[Title/Abstract]) AND ((type 2
diabetes[Title/Abstract]) OR T2DM[Title/Abstract] OR diabetes mellitus, type 2[MeSH Terms])
AND ((hepatocellular carcinoma[Title/Abstract]) OR HCC[Title/Abstract] OR liver
cancer[Title/Abstract] OR liver neoplasm*[Title/Abstract]).
```

Embase

```
(sglt2 inhibitor*:ti,ab OR sodium glucose cotransporter 2 inhibitor*:ti,ab OR
dapagliflozin:ti,ab OR empagliflozin:ti,ab OR canagliflozin:ti,ab OR ertugliflozin:ti,ab)
AND (type 2 diabetes:ti,ab OR t2dm:ti,ab) AND (hepatocellular carcinoma:ti,ab OR hcc:ti,ab
OR liver cancer:ti,ab).
```

Cochrane Library

```
(SGLT2 inhibitor OR sodium-glucose cotransporter-2 inhibitor OR dapagliflozin OR
empagliflozin OR canagliflozin OR ertugliflozin OR ipragliflozin) in Title Abstract Keyword
AND (type 2 diabetes OR T2DM) AND (hepatocellular carcinoma OR HCC OR liver cancer).
```

Final search date: 15 March 2026.
