# Supplementary material for: Association Between SGLT2 Inhibitor Use and Hepatocellular Carcinoma Risk in Type 2 Diabetes: A Systematic Review and Meta-Analysis
Source: Biomedicines. 2026 May 21;14(5):1168. doi: 10.3390/biomedicines14051168 (PMC13204993; doi:10.3390/biomedicines14051168)
Supplement: Supplementary file 1 [file biomedicines-14-01168-s001.zip › Supplementary_Table_S2_Extraction_Sheet_v9_0_FINAL.pdf]

Supplementary Table S2. Study-level extraction sheet

Study	Exposure definition	Comparator definition	Effect measure	Adjusted estimate	Covariates adjusted	Notes
Bea 2023	Incident SGLT2i use	DPP-4 inhibitor	HR	0.81 (0.67–0.98)	Demographics, liver risk, diabetes covariates	General T2DM cohort; Korean national claims data
Chou 2024	SGLT2i exposure	DPP-4 inhibitor	HR	0.42 (0.28–0.79)	Multivariable adjustment / matching	Comparator retained for pooling
Cho 2024	SGLT2i exposure in FLD + T2DM; CVH subgroup reported	Non-SGLT2i therapy	aHR	0.43 (0.29–0.63)	Multivariable adjustment	Korean HIRA FLD/T2DM cohort; chronic viral hepatitis subgroup available
Choi 2025	SGLT2i exposure	DPP-4 inhibitor retained from multiple active comparators	sHR	0.53 (0.30–0.93)	Active-comparator EHR cohort covariate set	United States/Korean EHR data; active-comparator framework
Kang 2026	SGLT2i exposure in viral hepatitis+T2DM	Non-SGLT2i / alternative antidiabetic therapy	sHR	0.77 (0.66–0.91)	Nationwide adjusted model	Korean nationwide viral hepatitis + T2DM cohort
Huynh 2023	Metformin + SGLT2i dual therapy	Metformin monotherapy	HR	0.43 (0.21–0.88)	Propensity score matching / adjusted analyses	TriNetX cirrhosis + T2DM cohort; metformin monotherapy comparator
