# Supplementary material for: Association Between SGLT2 Inhibitor Use and Hepatocellular Carcinoma Risk in Type 2 Diabetes: A Systematic Review and Meta-Analysis
Source: Biomedicines. 2026 May 21;14(5):1168. doi: 10.3390/biomedicines14051168 (PMC13204993; doi:10.3390/biomedicines14051168)
Supplement: Supplementary file 1 [file biomedicines-14-01168-s001.zip › Supplementary_Table_S3_LOO_v9_0_FINAL.pdf]

## Supplementary Table S3. Leave-one-out sensitivity analysis results (REML random-effects model)

| Study excluded | Pooled HR (95% CI) | $I^2$ (%) |
|---|---|---|
| Bea (2023) | 0.53 (0.39–0.72) | 62.2 |
| Chou (2024) | 0.62 (0.47–0.83) | 75.7 |
| Cho (2024) | 0.64 (0.48–0.84) | 70.8 |
| Choi (2025) | 0.59 (0.43–0.81) | 80.9 |
| Kang (2026) | 0.54 (0.39–0.74) | 64.1 |
| Huynh (2023) | 0.61 (0.45–0.81) | 78.7 |

All analyses used the restricted maximum likelihood (REML) estimator. The pooled estimate remained below 1.0 across all leave-one-out iterations, with a range of 0.53 to 0.64, indicating that no single study disproportionately influenced the overall result. Analyses were performed using R version 4.5.2 (R Foundation for Statistical Computing, Vienna, Austria) with the metafor package (version 4.8-0).
