# Supplementary material for: Association Between SGLT2 Inhibitor Use and Hepatocellular Carcinoma Risk in Type 2 Diabetes: A Systematic Review and Meta-Analysis
Source: Biomedicines. 2026 May 21;14(5):1168. doi: 10.3390/biomedicines14051168 (PMC13204993; doi:10.3390/biomedicines14051168)
Supplement: Supplementary file 1 [file biomedicines-14-01168-s001.zip › Supplementary_Table_S4_Sensitivity_v9_0_FINAL.pdf]

Supplementary Table S4. Sensitivity and subgroup analyses (REML random-effects model)

Analysis	k	Pooled HR (95% CI)	I^2 (%)	tau^2
Primary analysis	6	0.59 (0.45–0.77)	75.2	0.0740
Sensitivity: Excluding Huynh (2023)	5	0.61 (0.45–0.81)	78.7	0.0760
DPP-4i-restricted active-comparator	3	0.60 (0.39–0.92)	67.4	0.0923
Subgroup: Asian cohorts	4	0.61 (0.44–0.86)	85.0	0.0950
Subgroup: Non-Asian or multi-institutional cohorts	2	0.49 (0.31–0.76)	0.0	0.0000
Subgroup: CLD-enriched cohorts	3	0.56 (0.36–0.86)	74.6	0.1047
Subgroup: General T2DM cohorts	3	0.60 (0.39–0.92)	67.4	0.0923

Notes:

All analyses used the restricted maximum likelihood (REML) estimator. The 95% prediction interval for the primary analysis was 0.25–1.37 (t-distribution, df = k-2 = 4).

Asian cohorts: Bea (2023), Chou (2024), Cho (2024), Kang (2026). Non-Asian or multi-institutional cohorts: Choi (2025), Huynh (2023).

CLD-enriched cohorts: Cho (2024, FLD/T2DM and CVH-enriched cohort), Kang (2026, viral hepatitis+T2DM), Huynh (2023, cirrhosis+T2DM). General T2DM cohorts: Bea (2023), Chou (2024), Choi (2025).

The "General T2DM cohorts" subgroup comprises the same three studies as the DPP-4i-restricted active-comparator analysis, hence the identical pooled estimate and I^2 value.

E-value for the primary pooled estimate: 2.78; E-value for the confidence limit closest to the null: 1.92.

Analyses were performed using R version 4.5.2 (R Foundation for Statistical Computing, Vienna, Austria) with the metafor package (version 4.8-0).
