# Supplementary material for: Association Between SGLT2 Inhibitor Use and Hepatocellular Carcinoma Risk in Type 2 Diabetes: A Systematic Review and Meta-Analysis
Source: Biomedicines. 2026 May 21;14(5):1168. doi: 10.3390/biomedicines14051168 (PMC13204993; doi:10.3390/biomedicines14051168)
Supplement: Supplementary file 1 [file biomedicines-14-01168-s001.zip › Supplementary_Table_S5_MetaRegression_v9_0_FINAL.pdf]

Supplementary Table S5. Exploratory bivariable random-effects meta-regression with comparator type as moderator (REML)

CAUTION — Exploratory analysis only. This meta-regression is statistically underpowered ($k = 6$ studies; 1 moderator) and is reported here only to address Reviewer 3 Comment 1. Standard guidance recommends a minimum of approximately 10 studies per moderator coefficient for stable random-effects meta-regression. With $k = 6$, individual studies exert disproportionate leverage, the moderator coefficient confidence interval is wide, and the analysis is vulnerable to unstable estimates, ecological bias, and spurious associations. The results should not be used to infer comparator-specific effect modification.

Model specification

Model: $\log(HR)_i = \beta_0 + \beta_1 \cdot x_i + u_i + e_i$, with $x_i = 1$ if the retained primary contrast used a DPP-4 inhibitor comparator (Bea 2023, Chou 2024, Choi 2025) and $x_i = 0$ otherwise (Cho 2024 non-SGLT2i comparator; Kang 2026 non-SGLT2i comparator; Huynh 2023 metformin monotherapy comparator). Restricted maximum likelihood (REML) estimation was used to estimate the residual between-study variance τ^2, matching the estimator used for the primary intercept-only random-effects model reported in the main manuscript.

Regression coefficients

Model parameter	k	Estimate (95% CI)	z	p-value
Intercept (β_0): non-DPP-4 comparator reference	3	−0.581 (−1.009 to −0.154)	−2.67	0.008
Moderator (β_1): DPP-4 comparator vs non-DPP-4	3	+0.070 (−0.538 to +0.677)	+0.22	0.822
exp(β_0): predicted pooled HR (non-DPP-4 reference)	—	0.56 (0.36–0.86)	—	—
exp(β_1): ratio of HRs (DPP-4 vs non-DPP-4)	—	1.07 (0.58–1.97)	—	—

Heterogeneity summary

Heterogeneity metric	Value
τ^2 (residual; meta-regression)	0.099
τ^2 (intercept-only random-effects model)	0.074
Pseudo R^2 (variance in true effects explained by moderator)	0.0%
Q_E (test of residual heterogeneity)	29.15 (df = 4); $p < 0.001$
Q_M (omnibus test of moderator)	0.05 (df = 1); $p = 0.822$

Interpretation

The moderator coefficient β_1 was small in magnitude (+0.070 on the log scale) and statistically indistinguishable from zero ($p = 0.822$). The corresponding ratio of pooled hazard ratios (DPP-4 vs non-DPP-4 comparator) was 1.07 (95% CI 0.58–1.97), which spans roughly a halving to a doubling of the

relative effect; this confidence interval is too wide to support any inference about comparator-specific effect modification.

The pseudo R^2 was 0.0%, indicating that the DPP-4 versus non-DPP-4 comparator distinction did not explain any of the between-study variance in true effects in this small dataset. The residual τ^2 (0.099) was numerically higher than the intercept-only random-effects τ^2 (0.074), which is consistent with the well-recognized small-sample behavior of REML meta-regression when a moderator does not capture true between-study variance: addition of a non-informative moderator with $k = 6$ can spuriously inflate the estimated residual variance because the degrees of freedom available for variance estimation are reduced. This pattern further illustrates the instability of meta-regression at this sample size.

The residual heterogeneity test ($Q_E = 29.15$, df = 4, $p < 0.001$) confirmed that substantial unexplained between-study variation persisted after accounting for comparator type, consistent with the high I^2 (75.2%) reported for the intercept-only primary analysis. Multiple additional sources of clinical and methodological heterogeneity that could not be modelled simultaneously at $k = 6$ — including liver disease background (general T2DM vs FLD/CVH-enriched vs cirrhosis-enriched cohorts), HR versus subdistribution HR estimand, geographic region, and HCC ascertainment method — are likely to contribute to residual heterogeneity in ways that this single-moderator model cannot disentangle.

Why this analysis is not used as primary evidence

• Sample size: $k = 6$ studies for 1 moderator is below the conventional minimum of ~10 studies per moderator coefficient recommended for stable random-effects meta-regression.

• Confounded moderator structure: in the present dataset, the three studies using DPP-4 inhibitor comparators (Bea, Chou, Choi) coincide exactly with the three studies enrolling general T2DM populations, while the three non-DPP-4 studies (Cho, Kang, Huynh) all enrolled chronic liver disease-enriched populations. Comparator type and liver disease background are therefore perfectly collinear and cannot be separated.

• Wide confidence interval: the moderator CI (0.58–1.97) is consistent with effect modification ranging from a 42% reduction to a 97% increase in the relative HR, which provides no actionable information.

• Ecological-level reasoning: meta-regression evaluates between-study associations rather than within-study patient-level interactions and can produce spurious associations due to aggregation bias.

• Lack of patient-level data: residual confounding cannot be addressed at the meta-analytic level.

Methods

Effect sizes (y_i, v_i) were computed on the log hazard ratio scale from the six adjusted estimates and their 95% confidence intervals reported in the primary studies, applying the standard symmetric SE approximation $[\log(UCL) - \log(LCL)] / (2 \times 1.96)$ consistent with the main analysis. The mixed-effects meta-regression model was fitted with REML estimation. The test of the moderator (Q_M) was a Wald-type test of H_0: $\beta_1 = 0$. The residual heterogeneity test (Q_E) was a chi-squared test of H_0: $\tau^2_{residual} = 0$. The pseudo R^2 was computed as $\max(0, 1 - \tau^2_{residual} / \tau^2_{intercept\text{-}only})$. Analyses were performed using R version 4.5.2 (R Foundation for Statistical Computing, Vienna, Austria) with the metafor package (version 4.8-0). Results are reported here in support of Reviewer 3 Comment 1 and should be considered exploratory only.
